# Unilateral Calf Atrophy: A Case Series of Clinical and Electrodiagnostic Findings With a Review of the Literature

**DOI:** 10.7759/cureus.54710

**Published:** 2024-02-22

**Authors:** Lisa B Shields, Vasudeva G Iyer, Yi Ping Zhang, Christopher B Shields

**Affiliations:** 1 Norton Neuroscience Institute, Norton Healthcare, Louisville, USA; 2 Neurology/Clinical Neurophysiology, Neurodiagnostic Center of Louisville, Louisville, USA

**Keywords:** electromyography, nerve conduction studies, ultrasound, electrodiagnostic studies, tibial nerve, neurosurgery, calf atrophy, neurology

## Abstract

Unilateral calf atrophy may result from several medical conditions, such as lumbar radiculopathy, asymmetric myopathy/dystrophy, a Baker’s (popliteal) cyst leading to tibial nerve compression, and disuse atrophy. We present a case series of four patients with unilateral calf atrophy, including chronic neurogenic atrophy (benign focal amyotrophy, one patient), tibial nerve compression at the popliteal fossa by a Baker’s cyst (one patient), and disuse atrophy (two patients). All four patients underwent electrodiagnostic (EDX) studies, and two of them had denervation changes of the gastrocnemius. One patient underwent an ultrasound (US), which revealed a large cyst in the popliteal fossa causing compression of the tibial nerve. The differential diagnosis of unilateral calf atrophy as well as diagnostic techniques to confirm the underlying pathology are described. EDX and US studies are useful in differentiating between the varied conditions that may cause asymmetric calf muscle wasting.

## Introduction

The differential diagnosis of unilateral calf atrophy includes several medical disorders, including lumbosacral radiculopathy, asymmetric myopathy/dystrophy, late effect of lower extremity compartment syndrome, peroneal tendinopathy, Baker’s cyst resulting in tibial compression, amyotrophic lateral sclerosis, polymyositis, and disuse atrophy [1-3]. Of these etiologies of unilateral calf atrophy, lumbar radiculopathies of L5 and S1 are the most common ones [2]. In addition to nerve compression by a Baker’s cyst, other compressive pathologies include ganglion of the tibial nerve, tendinous arch of the soleus muscle, fibrous bands between the two heads of the gastrocnemius muscle, and tibial nerve tumors [4]. Differentiating between diverse causes of unilateral calf atrophy may prove challenging. In Baek et al.'s case report of unilateral calf atrophy and bilateral leg pain, the patient was diagnosed with polymyositis accompanying lumbar spinal stenosis and disc herniation [2]. As polymyositis involved the distal muscles of the lower extremities, it was often difficult to distinguish between polymyositis and spinal stenosis without electrodiagnostic (EDX) testing.

We present four unusual cases of unilateral calf atrophy: two with chronic tendinopathy (disuse atrophy), one due to tibial nerve compression at the popliteal fossa by a Baker’s cyst, and one due to chronic neurogenic atrophy (benign focal amyotrophy). We highlight the importance of EDX and ultrasound (US) studies in the investigation of unilateral calf atrophy.

## Case presentation

We discuss four cases of unilateral calf atrophy: two with chronic tendinopathy (disuse atrophy), one due to tibial nerve compression at the popliteal fossa by a Baker’s cyst, and one due to chronic neurogenic atrophy (benign focal amyotrophy) (Table 1).

**Table 1:** Cases of unilateral calf atrophy in this case series M: male; F: female; L: left; R: right; EDX: electrodiagnostic; CMAP: compound muscle action potentials; AH: abductor hallucis; US: ultrasound; ND: not done

Variables	Case #1	Case #2	Case #3	Case #4
Age (years)	54	78	82	63
Gender (M/F/)	M	F	M	F
Side (L/R)	L	R	F	L
Physical exam	Atrophy calf, weakness in plantar flexion	Weakness in plantar flexors foot, decreased pinprick sensation in plantar aspect foot, dorsal aspect forefoot	Knee reflexes decreased bilaterally, ankle reflexes absent bilaterally, decreased pinprick sensation up to ankle on R and up to mid-calf on L in dermatomal distribution	Atrophy calf, clawing foot, decreased pinprick sensation over plantar and dorsal aspects of foot
EDX findings	Progressive drop in amplitude of CMAP over AH in serial nerve conduction studies with normal motor conduction velocity	Drop in the amplitude of CMAPs over AH between stimulation at the ankle and popliteal fossa, slowing of motor conduction	Normal amplitude of CMAPs over AH, normal motor conduction velocity	Normal amplitude of CMAPs over AH, normal motor conduction velocity
US features	“Ground glass” appearance of gastrocnemius muscle	Baker’s cyst in the popliteal fossa adjacent to the tibial nerve	ND	ND
Diagnosis	Chronic neurogenic atrophy (benign focal amyotrophy)	Tibial nerve compression at the popliteal fossa by a Baker’s cyst	Chronic tendinopathy involving Achilles tendon (disuse atrophy)	Tendinopathy of Achilles tendon (disuse atrophy)
Follow-up	Lumbar decompression and fusion L4-5, no improvement of neuropathic symptoms 18 months postoperatively	Surgery to decompress the tibial nerve by resection of Baker’s cyst was recommended; the patient lost to follow-up	No follow-up	Diagnosed with Dupuytren’s contracture hand, may have underlying connective tissue disorder such as Ehlers-Danlos syndrome

Case #1

History and Physical Examination

**Figure 1:**
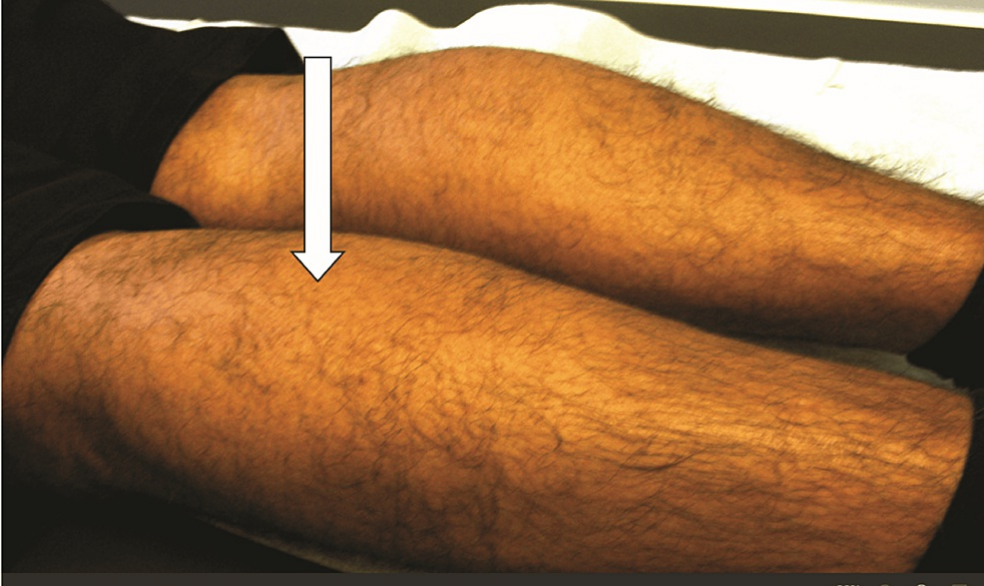
Atrophy of the left calf Case #1: atrophy of the left calf (arrow). The heads of the gastrocnemius on the left are flattened compared to the right

A 54-year-old male presented with a three-year history of lower back pain, which had been triggered by lifting a heavy box and, later, progressive weakness of the left calf muscle. He denied any pain in the left calf or foot. A physical exam revealed atrophy of the left calf with weakness of plantar flexion of the left foot (Figure 1). Strength in all other muscles was normal. The left Achilles tendon reflex was decreased compared to the right. The knee reflexes were symmetrical, and sensations were normal.

EDX Studies

**Figure 2:**
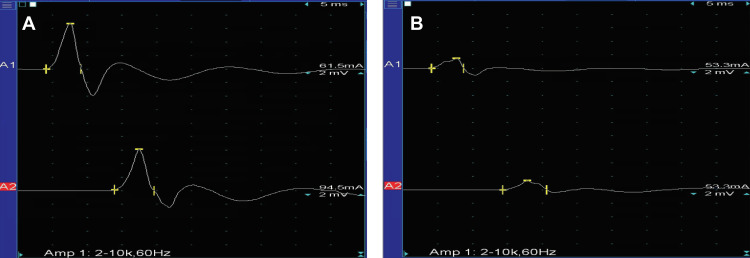
Nerve conduction velocity study Case #1: nerve conduction velocity study of the left tibial nerve. (A) An initial study was performed six years before the lumbar decompression and fusion. The amplitude of the compound muscle action potentials over the abductor hallucis with stimulation at the ankle was 6.8 mV; the amplitude of the compound muscle action potentials over the abductor hallucis with stimulation at the popliteal fossa was 6.00 mV (normal: ≥2 mV). The motor conduction velocity was 45.9 m/s (normal: ≥40 m/s). (B) Follow-up study one year after the lumbar surgery. The amplitude of the compound muscle action potentials over the abductor hallucis at the ankle was 1.48 mV; the amplitude of the compound muscle action potentials over the abductor hallucis at the popliteal fossa was 1.41 mV. The motor conduction velocity was 42.3 m/S. Note the significant decrease in amplitude of the compound muscle action potentials between Figure 2A and Figure 2B (6.8 mV vs. 1.48 mV, respectively). Recording site: abductor hallucis. A1: stimulus site: ankle. A2: stimulus site: popliteal fossa

Stimulation of the tibial nerve evoked an H reflex with prolonged latency on the left side (Figure 2A). Needle electromyography (EMG) showed positive sharp waves and occasional fibrillations in the left gastrocnemius more prominently in the lateral head, indicating denervation changes. The differential diagnoses included an S1 radiculopathy, benign focal atrophy, and perineuroma of the tibial nerve.

Radiological Findings, Surgical Intervention, and Follow-Up

A lumbar myelogram and post-myelographic CT scan revealed congenital stenosis as well as disc protrusion and foraminal stenosis at L3-4 and L4-5. A lumbar MRI performed four years later demonstrated multilevel degenerative changes with foraminal stenosis at L2-3, L3-4, and L4-5. The patient underwent lumbar decompression and fusion at the L4-5 interspace six years after the EDX studies, which led to minimal improvement in his lower back pain. The left calf atrophy persisted postoperatively. He also developed symptoms of sensory peripheral neuropathy and meralgia paresthetica bilaterally, presumably related to diabetes mellitus. 

**Figure 3:**
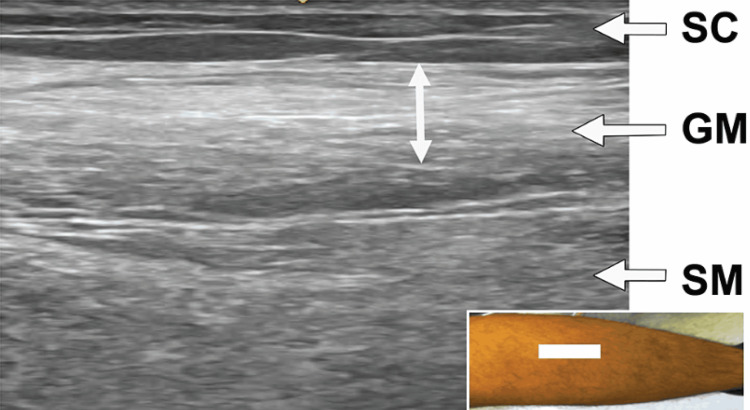
Ultrasound of gastrocnemius muscle with a “ground glass” appearance Case #1: ultrasound (long axis view) of the left gastrocnemius muscle (GM) demonstrating a “ground glass” appearance (vertical arrow showing white area). The soleus muscle (SM) is less affected SC: subcutaneous tissue

A needle EMG performed one year after his lumbar surgery showed fibrillations in the calf muscles bilaterally. An isolated motor unit was recruited on the left, and the motor unit recruitment was significantly decreased on the right. These findings suggested marked denervation of the left gastrocnemius and some denervation in the right gastrocnemius (Figure 2B). A US study of the left gastrocnemius demonstrated a “ground glass” appearance (Heckmatt grade 4; indicating very strong muscle echogenicity with near complete loss of distinct bone echo from the muscle in greater than 90% of the tissue) (Figure 3) [5]. No source of the compression of the tibial nerve was seen.

A diagnosis of unilateral calf atrophy from chronic neurogenic atrophy (benign focal amyotrophy) was made. At the last follow-up 18 months after his lumbar surgery, the patient did not show any improvement in his neuropathic symptoms.

Case #2

History and Physical Examination

A 78-year-old female presented with a four-year history of progressive atrophy of the right calf muscles as well as right knee pain and paresthesia of the feet bilaterally. She had undergone lumbar spine surgery 22 years earlier. Physical exam revealed significant weakness (MRC grade 3) of the plantar flexors of the right foot as well as decreased pinprick sensation over the plantar aspect of the right foot and dorsal aspect of the right forefoot.

EDX and US Studies

**Figure 4:**
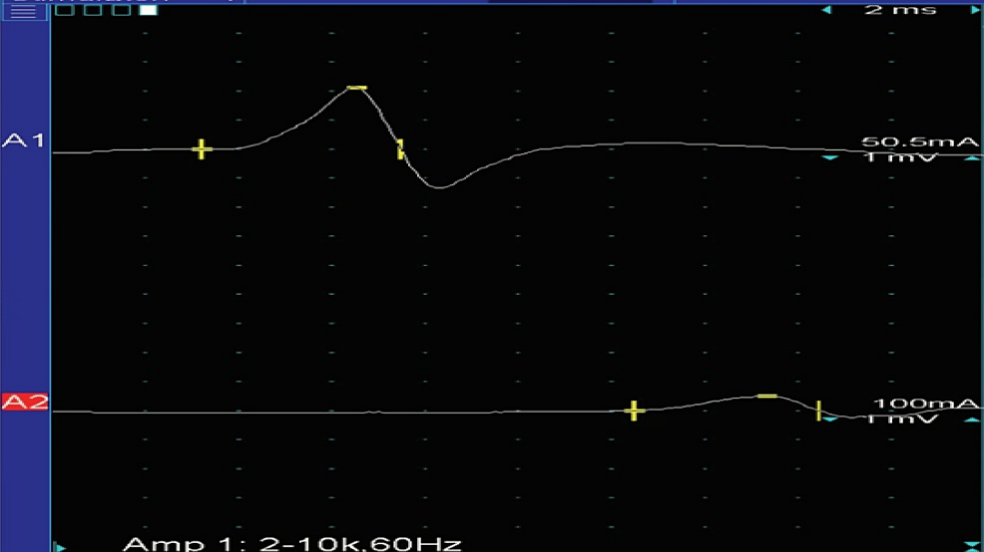
Nerve conduction velocity study Case #2: nerve conduction velocity study of the right tibial nerve. The amplitude of the compound muscle action potentials over the abductor hallucis with stimulation at the ankle was 2.17 mV; the amplitude of the compound muscle action potentials over the abductor hallucis with stimulation at the popliteal fossa was 0.51 mV (normal: ≥2 mV). The motor conduction velocity was 37.7 m/s (normal: ≥40 m/s). Note the drop in amplitude of the compound muscle action potentials over the abductor hallucis between stimulation at the ankle and the popliteal fossa (2.17/0.51 mV) and slowing of motor conduction (37.7 m/s as compared to a normal of ≥40 m/s). Recording site: abductor hallucis A1: stimulus site: ankle. A2: stimulus site: popliteal fossa

Needle EMG showed positive sharp waves in the right gastrocnemius with significantly decreased motor unit recruitment with large polyphasic units. Upon right tibial nerve stimulation, the H-reflexes were absent, the compound muscle action potential (CMAP) was of low normal amplitude, and motor conduction velocity was decreased (Figure 4). The sensory nerve action potentials (SNAP) were also absent. Motor unit morphology and recruitment pattern suggested chronic denervation and reinnervation in the right gastrocnemius.

**Figure 5:**
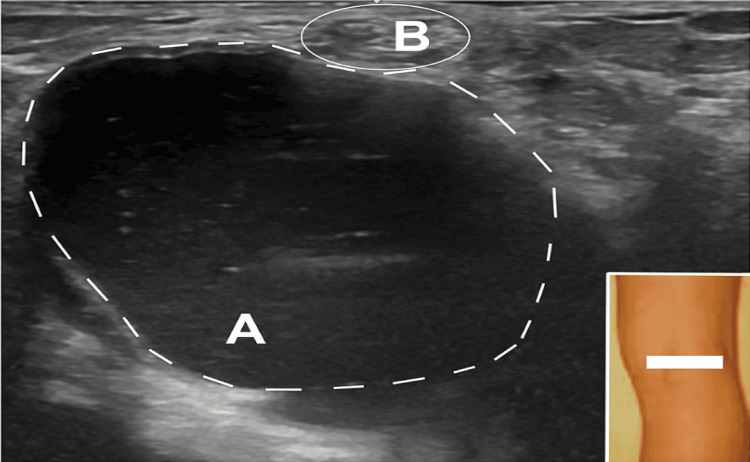
Ultrasound of the popliteal fossa Case #2: ultrasound (short axis view) at the popliteal fossa showing (A) Baker’s cyst (surrounded by white dashes) and (B) tibial nerve (encircled by an oval)

A US study demonstrated a large cyst in the popliteal fossa, adjacent to the tibial nerve (Figure 5). The final diagnosis was tibial nerve compression at the popliteal fossa by a Baker’s cyst.

Follow-Up

Surgical intervention was recommended to decompress the tibial nerve by resection of the Baker’s cyst. This surgery can be safe if the nerve conduction (through the tibial and peroneal nerves) is monitored during the procedure. The patient moved out of state to be closer to family members and hence was lost to follow-up.

Case #3

History, Physical Examination, and Radiological Findings

An 82-year-old male presented with a history of decreased size of the right calf muscle and pain in the right Achilles tendon. On physical exam, wasting of the right calf muscles was noted. He was unable to stand on his right toes as well as his left toes. There was also significant pain in the right Achilles tendon. The knee reflexes were decreased bilaterally, and the ankle reflexes were not elicitable bilaterally. Decreased pinprick sensation up to the ankle on the right and up to the mid-calf on the left in a dermatomal distribution was observed. 

A lumbar MRI demonstrated no evidence of central canal narrowing or lateral recess or foraminal narrowing at the L5-S1 level. There was no indication of S1 nerve root compression on the right.

EDX Studies

**Figure 6:**
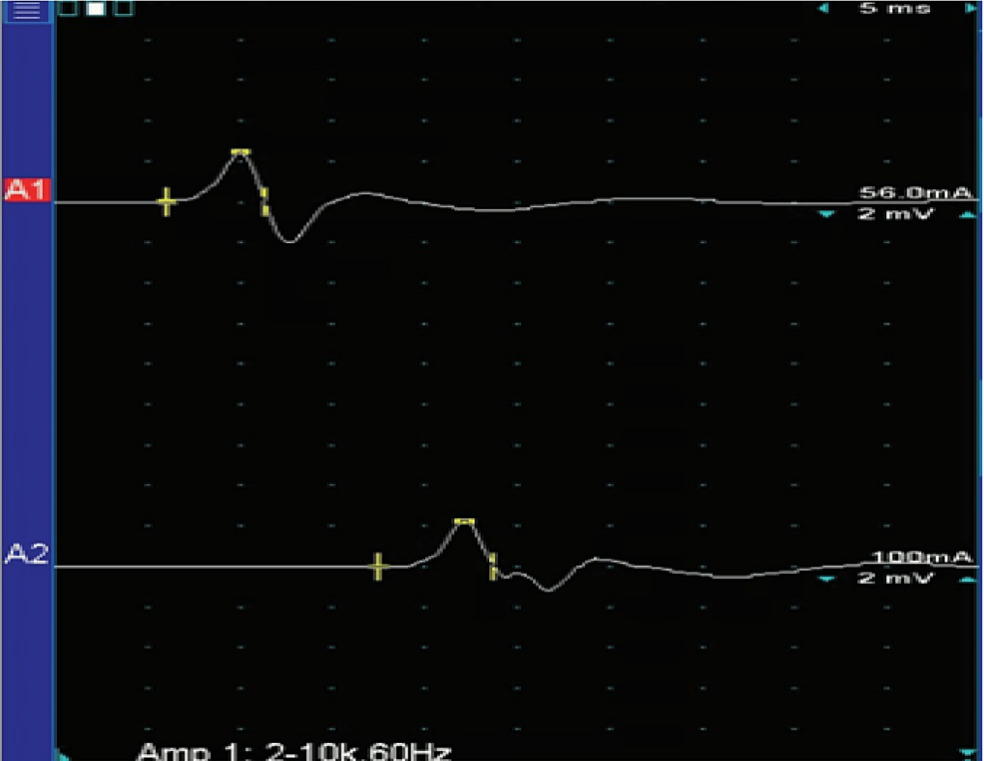
Nerve conduction velocity study Case #3: nerve conduction velocity study of the right tibial nerve. The amplitude of the compound muscle action potentials over the abductor hallucis with stimulation at the ankle was 2.47 mV; the amplitude of the compound muscle action potentials over the abductor hallucis with stimulation at the popliteal fossa was 2.32 mV (normal: ≥2 mV). The motor conduction velocity was 40.0 m/s (normal: ≥40 m/s). The amplitude of the compound muscle action potentials over the abductor hallucis was normal, and the motor conduction velocity was normal. Recording site: abductor hallucis A1: stimulus site: ankle. A2: stimulus site: popliteal fossa

Needle EMG did not show denervation or reinnervation changes in the right gastrocnemius. Nerve conduction velocity studies of the right tibial nerve revealed a normal amplitude of the compound muscle action potentials over the abductor hallucis, and the motor conduction velocity was normal (Figure 6). Nerve conduction abnormalities were suggestive of mild motor sensory neuropathy related to diabetes mellitus. The final diagnosis was right calf muscle atrophy likely secondary to chronic tendinopathy involving the Achilles tendon (disuse atrophy).

Case #4

History, Physical Examination, and Radiological Findings

A 63-year-old female presented with weakness of the left lower extremity and atrophy of the left calf muscle. She underwent surgery for rupture of the tibialis anterior and extensor hallucis longus tendons and lengthening of the Achilles tendon, after which she reported difficulty with ambulation and left calf atrophy. On exam, significant atrophy of the left calf muscles and clawing of the left foot were observed. Weakness of dorsiflexion, eversion, and plantar flexion of the left foot was noted. There was also decreased pinprick sensation over the plantar and dorsal aspects of the left foot.

Shoulder, knee, and ankle MRI scans all supported extensive tendinopathy. The ankle MRI revealed diffuse thickening of the tibialis anterior and posterior, extensor hallucis longus, and Achilles tendons as well as high-grade tearing of the peroneal longus and brevis tendons.

EDX Studies

**Figure 7:**
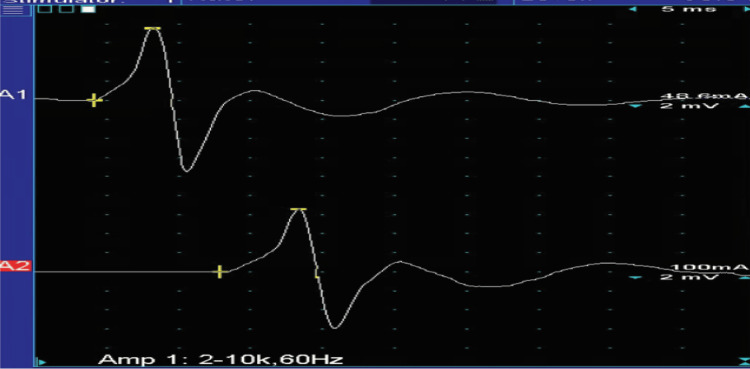
Nerve conduction velocity study Case #4: nerve conduction velocity study of the left tibial nerve. The amplitude of the compound muscle action potentials over the abductor hallucis with stimulation at the ankle was 7.63 mV; the amplitude of the compound muscle action potentials over the abductor hallucis with stimulation at the popliteal fossa was 6.67 mV (normal: ≥2 mV). The motor conduction velocity was 42.0 m/s (normal: ≥40 m/s). The amplitude of the compound muscle action potentials over the abductor hallucis was normal, and the motor conduction velocity was normal. Recording site: abductor hallucis A1: stimulus site: ankle. A2: stimulus site: popliteal fossa

Needle EMG did not show denervation or reinnervation changes in the left gastrocnemius. Nerve conduction velocity studies of the left tibial nerve demonstrated a normal motor conduction velocity (≥40 m/s). The amplitude of the compound muscle action potentials over the abductor hallucis was normal (≥2 mV) (Figure 7). The final diagnosis was left calf muscle atrophy secondary to tendinopathy of the Achilles tendon (disuse atrophy).

Follow-Up

The patient was subsequently diagnosed with Dupuytren’s contracture of the right hand. Given the involvement of several tendons including a history of numerous tendon ruptures, an underlying connective tissue disorder such as Ehlers-Danlos syndrome was suspected as her mother also had a history of hand deformities.

## Discussion

The initial step in the investigation of asymmetric lower extremity muscle wasting involves performing a focused neurological examination of the lower extremities. Several diagnostic tests may also be performed to elucidate the cause of the unilateral calf atrophy (Tables 2, 3) [1]. A lumbar MRI scan is usually the initial diagnostic test in the evaluation of unilateral calf atrophy. If a Baker’s cyst is palpated or suspected, a knee MRI scan should be performed. An EMG/NCV study of the lower extremities may help differentiate lumbar radiculopathy from peripheral neuropathy. In cases of disuse atrophy from muscle or tendon injury, the EDX studies tend to be normal. Additionally, this test may localize the site of nerve involvement [4]. Serial EDX studies are often advantageous to assess the effectiveness of therapy [6]. The inexpensive, noninvasive, and readily available US may detect a mass such as a Baker’s cyst that is compressing the tibial nerve, resulting in asymmetric calf atrophy [3]. Additional testing modalities include a cervical or thoracic MRI [7] and an ankle MRI. A muscle biopsy may also be performed if the diagnosis is not clear. Detecting an elevated creatine kinase (CK) level in the blood may indicate muscle injury or disease.

**Table 2:** Differential diagnosis of unilateral calf atrophy

Differential diagnosis
Lumbar radiculopathy [1-3]
Asymmetric myopathy/dystrophy [1]
Lower extremity compartment syndrome (late effect) [1,6-11]
Tendo Achilles tendinopathy [12]
Amyotrophic lateral sclerosis [1]
Baker’s (popliteal) cyst causing tibial nerve compression [3,4,6-10,13-15]
Polymyositis [2]
Disuse atrophy [16]
Neurogenic atrophy [17]

**Table 3:** Diagnostic tests to investigate unilateral calf atrophy MRI: magnetic resonance imaging; EMG: electromyography; NCV: nerve conduction velocity study

Diagnostic tests
Lumbar MRI [1-3,8]
Knee MRI (if Baker’s cyst is palpated or suspected) [4,8,9]
EMG/NCV of the lower extremities [3,4,6-9,12-15,18]
Ultrasound [3,9]
Laboratory studies: total creatine kinase [1,2,12]
Cervical/thoracic MRI [1,19]
Ankle MRI [1]
Muscle biopsy [1]

Akechi et al. have reported a case of right lower limb atrophy associated with multiple glomus tumors of the lower extremity [12]. The patient complained of a 40-year history of chronic right leg pain. Needle EMG revealed no pathological neurogenic or myogenic changes. The authors suggested that glomus tumors of the leg may cause disuse atrophy. Disuse atrophy refers to the loss of skeletal muscle mass that may occur following limb immobilization, bed rest, spinal cord injury, and injuries to tendons and muscles. The degree of atrophy is dependent on the patient’s age as well as the physiological function of the muscle [9]. The mechanism of disuse atrophy involves lack of use, reduced neural activity, and decreased protein synthesis [9]. Mobilizing the joint and resistance exercises may attenuate muscle mass loss and increase strength after disuse atrophy. In the present series, two patients developed unilateral calf atrophy, which was attributed to disuse. Case #4 underwent surgery for a rupture of the tibialis anterior and extensor hallucis longus tendons and lengthening of the Achilles tendon after which she had ambulation difficulties. The unilateral calf atrophy was a sequela of these mobility challenges. Disuse in Case #3 was likely due to chronic tendinopathy of the Achilles tendon that led to the unilateral calf atrophy.

Neurogenic atrophy refers to the loss of muscle mass and function that results from injury or disease of the peripheral nervous system [10]. Myofiber denervation may lead to target or targetoid myofibers, usually involving type I fibers, which are most frequently observed in the gastrocnemius muscle. Skeletal muscle denervation causes activation of FOXO and NF-kB transcription factors, which includes a set of “atrogenes” that increase protein degradation through both ATP-dependent ubiquitin-proteasomal degradation and lysosomal/autophagic degradation [10]. Benign focal amyotrophy disorders (BFADs) are a clinically heterogeneous group of disorders that cause weakness and atrophy of the upper and/or lower extremities [11]. They are characterized by restricted limb involvement, absence of upper motor neuron signs, and slow progression followed by disease stabilization. Felice et al. reported eight cases of benign calf amyotrophy, a variant of BFAD [18]. These patients developed progressive calf muscle weakness and wasting. The electromyographic features were consistent with a chronic neuropathic disorder with more diffuse lower limb involvement. In the current case series, the unilateral calf atrophy in Case #1 was attributed to chronic denervation atrophy as confirmed by the EDX studies with marked denervation of the left gastrocnemius muscle.

“Baker’s cyst” is a synovial cyst in the popliteal fossa, and it is named after Dr. William Morrant Baker, who in 1877 published eight cases with three illustrations [20]. Baker noted that osteoarthritis was an underlying condition in several of these cases. A Baker’s (or popliteal) cyst is caused by enlargement of the gastrocnemio-semimembranosus bursa [3,6,13-15]. Baker’s cysts cause pain with activity and may lead to pseudo-thrombophlebitis (formed by extravasation of fluid into compartments of the calf), compartment syndrome, and entrapment neuropathy of the tibial, common peroneal, or sural nerves [3,4,6,13,14,16,17]. The sciatic nerve divides into the tibial and common peroneal nerve proximal to the popliteal fossa after which the tibial nerve passes along the posterior aspect of the popliteal fossa, supplying the calf muscles and providing an anastomotic branch to the sural nerve [13,17]. The tibial nerve subsequently continues as the posterior tibial nerve from the distal aspect of the popliteus muscle, passing along the posterior leg. Due to its most superficial and medial location within the popliteal fossa, the tibial nerve is frequently compressed by a Baker’s cyst [18]. The neuropathy may be due to either the rupture of the Baker’s cyst or direct pressure from an intact cyst [3,13]. Tibial nerve entrapment by a Baker’s cyst may present with signs and symptoms such as atrophy of the gastrocnemius muscle, paresthesia, pain, or numbness [3,14,18]. 

Certain knee pathologies such as arthritis, meniscal tears, and gout may predispose patients to the development of a Baker’s cyst due to disruption of the synovial fluid dynamics resulting in fluid accumulation within the knee tendon bursa [18]. Several diagnostic modalities including EDX studies, MRI (knee and spine), and US may help differentiate conditions that comprise the differential diagnosis, including a Baker’s cyst (ruptured or intact), disc lesion, thrombophlebitis, popliteal artery or vein aneurysms, tumor, or gastrocnemius tear [6,13,14,17]. The treatment involves either conservative management (rest, application of ice, leg elevation, analgesic and anti-inflammatory medications), aspiration of the cyst, intraarticular steroid injection, or open resection of the cyst [6,13,14]. A synovectomy is reserved for refractory and recurrent cases. As Baker’s cysts commonly recur, long-term close monitoring is necessary in these patients [18].

Several studies have reported nerve compression due to a Baker’s cyst [3,4,6,13-17,19,20] (Table 4). Of the 12 reported cases with tibial or both tibial and peroneal nerve compression, the compression involved the former in eight cases and the latter in four cases. Most patients were diagnosed with rheumatoid arthritis (RA), psoriatic arthritis, or degenerative joint disease. The physical examination findings revealed the particular nerve compressed by the cyst, specifically, the tibial, common peroneal, or a combination of these. The treatment pursued varied among these patients and included conservative management (analgesic medications, bed rest, gabapentin, transcutaneous electrical nerve stimulation, electrical stimulation therapy, physical therapy), which led to symptom improvement, as well as intra-articular injections of the knee joint, needle aspiration of the cyst, and open surgery with cyst resection.

**Table 4:** Cases of nerve compression due to Baker’s cyst reported in the literature RA: rheumatoid arthritis; IAI: intra-articular injections; DJD: degenerative joint disease; PsA: psoriatic arthritis; TKA: total knee arthroplasty

Study	Number of patients/nerves involved	Gender/ age in years	Physical examination	Arthritis	Treatment/outcome
Nakano, 1978 [10]	5 patients (1: common peroneal N; 1: sural nerve)	3M, 2F/51-66	3 patients (motor weakness anterior tibial, extensor hallucis longus, extensor digitorum longer and brevis, peroneal group, gastrocnemius soleus, posterior tibial, intrinsic foot muscles; light touch and pinprick deficits; absent or asymptomatic ankle jerk affected limb)	RA: 4 patients	IAI: 4 patients: the return of motor sensory function
	3 (common peroneal and posterior tibial nerves)		1 patient (foot drop, weakness anterior tibial, extensor hallucis longus, extensor digitorum longus and brevis, peroneus longus and brevis, diminished light touch and pinprick sensation lateral leg/dorsum foot; 1 patient (loss of light touch and pinprick sensation posterior lateral leg and foot	Trauma: 1 patient	Synovectomy: 1 patient
Zygmunt et al., 1982 [15]	2 patients (1: posterior tibial nerve; 1: common peroneal nerve)	M/55, M/68	1 patient (knee pain, decreased pinprick sensation, and sense of touch plantar 4th/5th toe: posterior tibial nerve)	RA: 1 patient; seronegative	1 patient (pain resolved without medications or surgery; 1 year later, continued reduced sensation of the foot)
			1 patient (pinprick sensation and diminished sensory touch along the peroneal nerve area)	RA: 1 patient	1 patient (cyst resection, sensory function improved immediately post-surgery)
Kashani et al., 1985 [13]	1 (posterior tibial nerve)	M/60	Absent toe flexors, 1+ ankle deep tendon reflex; sensation to touch, pain, temperature absent on plantar foot	Degenerative joint disease	Bed rest, analgesics, IAI, knee joint aspiration; within 2 weeks, lower extremity sensation improved
DiRisio et al., 1994 [7]	1 (posterior tibial nerve)	M/50	Difficulty with toe-walking; the collapse of the plantar flexed foot; normal sensation; absent ankle jerk	No arthritis	Synovectomy and decompression of branch of the posterior tibial nerve to the medial head of gastrocnemius; improved plantar flexion at 3 months postop
Sun et al., 1995 [4]	1 (tibial nerve)	M/32	Unable to flex toes, difficulty abducting toes; ankle reflex reduced; sensory deficit light touch and pinprick over the plantar surface foot that extended to medial ankle; manual compression of popliteal fossa produced dysesthesia in toes	No arthritis	Surgical decompression of popliteal cyst; 2 months postop, pain had decreased, some recovery of toe flexion and abduction; no improvement in sensation
Dash et al., 1998 [6]	1 (posterior tibial nerve)	M/53	Loss of sensation light touch and pain anterior half of the sole; atrophy at flexor hallucis brevis	PsA	IAI knee joints; improved foot symptoms
Lee et al., 2000 [9]	1 (posterior tibial nerve)	F/64	Pain/increased sensation plantar aspect foot, weak flexion big toe, decreased ankle jerk	RA	Repeated needle aspiration of cyst not helpful; underwent open surgery; pain resolved postoperatively, continued to experience numbness
Ji et al., 2007 [8]	1 (posterior and common peroneal nerves)	F/58	Calf atrophy, foot drop; Grade 4 dorsiflexion and Grade 2 plantar flexion of the ankle joint, Grade 3 eversion and Grade 2 inversion of the foot	No arthritis	Arthroscopic partial medial meniscectomy to decompress Baker’s cyst; cyst recurred 6 weeks postoperatively: then open resection; 1-year follow-up: improved motor/sensory function, no recurrence
Moon et al., 2013 [3]	1 (posterior tibial nerve)	49/M	Grade IV muscle strength anile plantar flexors, Grade I muscle strength great and little toe abductors; paresthesia sole	RA	Symptoms improved with nonoperative treatment (gabapentin 1200 mg, transcutaneous electrical nerve stimulation, electrical stimulation therapy, physical therapy)
Moyad, 2015 [14]	1 (tibial nerve)	F/65	Firm and tender posterior compartment of the leg; sensory deficit over the foot	No arthritis	Aspiration of the cyst; revision TKA with decompression of Baker’s cyst; pain resolved and sensory deficit decreased within 3 months postoperatively, posterior leg compartment remained decompressed
Present case, 2024	1 (tibial nerve)	F/78	Weakness in plantar flexors of the foot, decreased pinprick sensation over the plantar aspect of the foot and dorsal aspect of the forefoot	Arthritis	Lost to follow-up

EDX studies were performed in two cases that involved compression of solely the tibial nerve by a Baker’s cyst (Table 3). In the report by Sun et al. on tibial motor mononeuropathy, physical examination revealed an inability to flex the right toes and difficulty in abducting the right toes as well as a reduced right ankle reflex [4]. Light touch and pinprick were reduced over the plantar surface of the patient's right foot that extended to the medial ankle. EDX studies demonstrated a greater than 80% decline in amplitude of the motor “M” potential between the right ankle and popliteal fossa stimulating sites indicating a conduction block. A needle EMG study of the abductor hallucis showed occasional positive sharp waves without fibrillations, and no voluntary motor unit potentials were recruited. Surgical decompression of the Baker’s cyst was performed. In Moyad’s case involving tibial nerve neuropathy due to a Baker’s cyst, the cyst developed secondary to polyethylene wear 16 years after a total knee replacement [14]. EDX studies revealed a moderately severe tibial neuropathy likely at the popliteal fossa or distal to it with a chronic tibial nerve peripheral compressive mononeuropathy. A surgical decompression of the Baker’s cyst was performed. Case #2 in the present series had a history of arthritis; the physical examination indicated a tibial nerve neuropathy that was confirmed by EDX and US studies, and the treatment course was unknown as the patient was lost to follow-up.

## Conclusions

Lumbar spinal and foraminal stenosis is the most common cause of unilateral calf atrophy. Asymmetric calf muscle wasting is rarely caused by a Baker’s cyst, which can be easily confirmed by an MRI of the knee. EDX studies combined with US are highly effective methods to differentiate the causes of unilateral calf atrophy. Physicians should be cognizant of the diverse causes of unilateral calf atrophy.
